# Synergistic photothermal therapy of esophageal cancer using Pt@MOF@PSs nanozymes

**DOI:** 10.3389/fbioe.2026.1729547

**Published:** 2026-02-05

**Authors:** Yuhang Shang, Yujie Zhao, Ran Ding, Xinyue Gao, Qi Li, Ziyi Li, Xinglan An

**Affiliations:** 1 Key Laboratory of Organ Regeneration and Transplantation of the Ministry of Education, The First Hospital of Jilin University, Jilin University, Changchun, China; 2 Department of Intensive Care Unit, The First Affiliated Hospital of Jiamusi University, Jiamusi, China; 3 Candidate State Key Laboratory of Pharmaceutical Biotechnology and Jiangsu Key Laboratory of Molecular Medicine, Nanjing University Medical School, Nanjing, China

**Keywords:** esophageal cancer, IR780, metallic organic framework, nanozyme, photothermal therapy

## Abstract

Globally, esophageal cancer (EC) is the seventh most commonly diagnosed cancer and the sixth leading cause of cancer-related death. However, its treatment remains challenging due to significant obstacles. Photothermal therapy (PTT), a minimally invasive technique, has emerged as a promising method for tumor ablation. However, its efficacy is limited by low photothermal conversion efficiency and poor tissue penetration. To address these limitations, this study developed a metal-organic framework (MOF)-based nanozyme for the treatment of EC. In this system, the dye IR780, used for photothermal conversion, was encapsulated in liposomes and anchored onto the MOF nanozyme, resulting in a Pt@MOF@PSs construct that improved the aqueous stability of IR780. This multifunctional nanozyme showed tumor-targeting and synergistic therapeutic effects. After passive accumulation in EC tissues, Pt@MOF@PSs suppressed hypoxia and promoted reactive oxygen species (ROS) production by using the high H_2_O_2_ levels typical of the tumor microenvironment. The PTT activity of Pt@MOF@PSs was confirmed by its significant temperature increase and upregulation of heat shock protein 70 after irradiation with an 808 nm near-infrared laser. These features facilitated the effective modulation of the resistant tumor microenvironment, induced localized hyperthermia, exerted potent cytotoxicity against esophageal squamous carcinoma cells (ESCs), and suppressed EB tumor progression. These findings highlight Pt@MOF@PSs as a promising therapeutic option, integrating hypoxia relief, ROS generation, and PTT for improved therapeutics against EC.

## 1 Introduction

Esophageal cancer (EC) arises from the epithelial lining of the esophagus, most commonly presenting as squamous cell carcinoma or adenocarcinoma (Luo et al., 2021; Kato et al., 2021). It is one of the most common cancers worldwide, characterized by a high incidence and mortality rates and poor prognosis. The reported 5-year survival rate remains unsatisfactory, with approximately 46.7% for patients with locally advanced disease and only 4.8% for those with distant metastases (Mai et al., 2025). Current treatments for EC mainly include surgical resection, chemotherapy, radiotherapy, immunotherapy, and targeted therapy. However, around 40% of patients are not suitable for surgical intervention at the time of diagnosis, and chemotherapy alone has shown limited clinical benefits (Uhlenhopp et al., 2020). Moreover, insufficient therapeutic efficacy frequently results in incomplete eradication of tumor cells, which can lead to relapse and metastasis (Boone et al., 2009; Gupta et al., 2024). Recently, advanced techniques such as photothermal therapy (PTT), photodynamic therapy, and sonodynamic therapy have attracted attention as promising options for destroying malignant tumors, with PTT emerging as a potential treatment for EC (Li et al., 2024a; Li et al., 2021a). Among them, PTT has attracted increasing interest as a minimally invasive therapeutic method that eradicates malignant cells by generating localized hyperthermia (Huang et al., 2025; Chen et al., 2023; Bian et al., 2021). Photodynamic therapy (PDT) holds particular significance for esophageal cancer due to the unique pathological features of EC tumors. The hypoxic and acidic TME of esophageal squamous cell carcinoma (ESCC) often compromises conventional therapies, but PDT can directly target these regions by generating cytotoxic reactive oxygen species (ROS) under light irradiation. The Pt@MOF@PSs nanozyme enhances this effect by leveraging its peroxidase-like activity to decompose endogenous H_2_O_2_ into ROS, thereby amplifying oxidative stress within tumor cells. This nanozyme-mediated PDT not only suppresses hypoxia-induced resistance but also synergizes with PTT to induce immunogenic cell death, offering a dual-mode strategy to address the high recurrence rates and poor prognosis associated with advanced EC. The mechanism involves the conversion of near-infrared (NIR) light energy into thermal energy through the excitation of photothermal transduction agents (PTAs). In clinical and preclinical studies, PTAs usually raise intratumoral temperatures >50 °C under NIR irradiation, which effectively induces necrosis of tumor cells (Wen et al., 2022; Li et al., 2021b; Li et al., 2022). Recently, various nanomaterials have been developed as PTAs due to their remarkable photothermal conversion efficiency, stability, and good biocompatibility. These nano-PTAs are generally divided into two groups: organic PTAs, which consist of dye molecules and polymeric materials, and inorganic PTAs, including noble-metal nanostructures, magnetic nanoparticles, and semiconducting agents. Among organic PTAs, heptamethine cyanine IR780 iodide has emerged as a potent candidate due to its strong absorption in the NIR range (Li et al., 2024b) (Tsai et al., 2025). However, the application of IR780 in tumor therapy is limited by poor water solubility, a tendency to aggregate, and unstable photothermal conversion. Moreover, IR780 shows rapid clearance from circulation, a short half-life, and a lack of tumor-specific targeting capacity, all of which limit its translational potential and necessitate improvements in delivery methods. To address these limitations, efforts have increasingly focused on developing nanoplatforms capable of transporting and stabilizing IR780 (Refaat et al., 2023). Various nanocarrier strategies have been explored, including polymeric encapsulation, mesoporous silica, and liposomal vehicles. For example, Zhang and colleagues designed virus-like particles as carriers for IR780 (Lu et al., 2021), achieving effective tumor ablation through combined PTT and photodynamic therapy (PDT) in breast cancer models. Similarly, Qiao et al. fabricated IR780@tLyP-1-MGF6 nanoparticles that targeted PTT with improved therapeutic performance (Yang et al., 2025). Despite these advances, the therapeutic efficacy of IR780-based systems remains limited by the hostile tumor microenvironment (TME), which often shows therapy resistance.

The TME is characterized by hypoxia, mild acidity, and reductive conditions, all of which can reduce the efficacy of chemotherapy, radiotherapy, PDT, PTT, and other treatments (Pang and Wu, 2025). The abnormal biochemical traits of the TME have emerged as a promising strategy to improve therapeutic outcomes (Xing et al., 2024). Enzymes that use excess H_2_O_2_ to generate oxygen or ROS have shown potential in relieving hypoxia and reshaping the TME. However, natural enzymes are susceptible to environmental factors, such as temperature and pH, and their catalytic activity is limited to narrow operational ranges. In comparison, nanozymes, engineered nanostructures with enzyme-mimicking catalytic properties, offer higher stability, lower production costs, and easier storage (Yang et al., 2024; Luo et al., 2025; Jiang et al., 2019). Based on their catalytic mechanisms, nanozymes are typically classified into peroxidase (POD)-like, catalase (CAT)-like, SOD-like, and other types (Zhang et al., 2024a; Zhang et al., 2024b). Recent studies have highlighted the growing interest in multifunctional nanozyme platforms that integrate catalytic activity with photothermal or photodynamic modalities to overcome tumor microenvironment–associated therapeutic resistance, thereby enabling synergistic cancer treatment strategies (Liu et al., 2022; Shubhra, 2023; Guo et al., 2022). Among these, POD-mimicking nanozymes decompose H_2_O_2_ to produce toxic ROS, which promotes cancer cell apoptosis (Fang et al., 2024). Some metal-organic framework (MOF)-based nanozymes with POD-like activity have been developed, showing promising antitumor effects (Wang et al., 2025; Deng et al., 2024). Similarly, nanozymes with CAT-like activity can convert H_2_O_2_ into molecular oxygen, helping to reduce hypoxia within the TME (Xu et al., 2022). Recently, noble-metal nanozymes with CAT-like behavior have shown effective suppression of tumor growth. Therefore, designing nanozymes with both POD- and CAT-like catalytic activities is a highly promising approach for synergistically modulating the TME and improving therapeutic efficacy against cancer.

Importantly, the synergistic relationship between nanozyme activity and PTT is key to enhancing therapeutic outcomes. The CAT-like activity of nanozymes alleviates hypoxia, which is known to confer resistance to hyperthermia by reducing the sensitivity of tumor cells to heat. Simultaneously, the POD-like activity generates ROS that not only directly induce apoptosis but also sensitize tumor cells to PTT by damaging cellular components and promoting heat shock protein downregulation. This dual modulation of the TME ensures that PTT is more effective, as the nanozyme activity preconditiones the tumor cells for thermal ablation, leading to improved cytotoxicity and reduced relapse risk.

**SCHEME 1:**
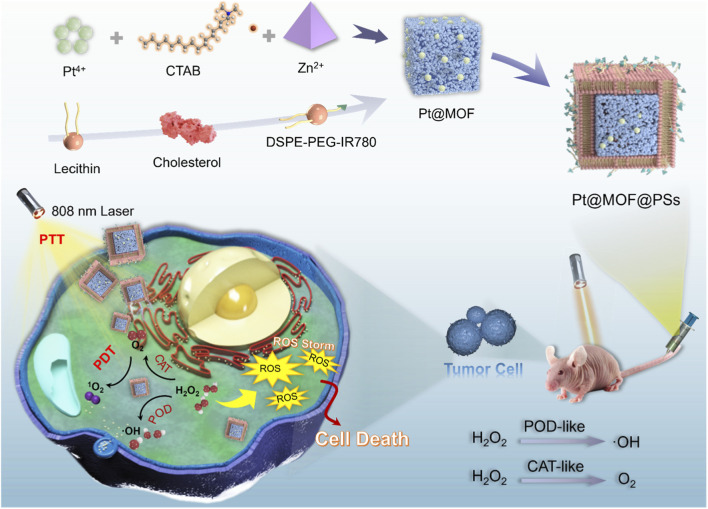
Schematic diagram of Pt@MOF@PSs and the mechanism of EC treatment.

Based on these considerations, a multifunctional nanoplatform called Pt@MOF@PSs was developed for PTT and PDT of EC under 808 nm NIR irradiation (Scheme 1). The system was fabricated through a multi-step process: first, a Pt-MOF nanozyme was synthesized using H_2_PtCl_6_ as the metal precursor; then, the photothermal dye IR780 was loaded into a liposomal structure composed of 1,2-distearoyl-sn-glycero-3-phosphocholine (DSPC) and cholesterol; finally, the Pt-MOF was incorporated into this IR780-loaded liposome to form Pt@MOF@PSs. This hybrid nanozyme showed dual catalytic activity, mimicking both POD and CAT enzymes. In tumor-like solutions rich in H_2_O_2_, Pt@MOF@PSs mediated two simultaneous reactions: converting H_2_O_2_ into cytotoxic ROS and decomposition of H_2_O_2_ into oxygen, thus reducing hypoxia and reprogramming the reductive TME. Simultaneously, the nanoplatform showed a strong photothermal conversion efficiency, generating localized hyperthermia and upregulating heat shock protein 70 (HSP70) as well as generating abundant ROS under PDT. Moreover, the Pt@MOF@PSs showed excellent biocompatibility and primarily accumulated in tumor tissues through the enhanced permeability and retention (EPR) effect. Both *in vitro* and *in vivo* analyses confirmed its high photothermal and catalytic effectiveness. The Pt@MOF@PSs nanoplatform showed significant antitumor activity by synergistically modifying the TME and providing PTT and PDT, presenting a promising approach for EC treatment.

## 2 Result and discussion

**FIGURE 1:**
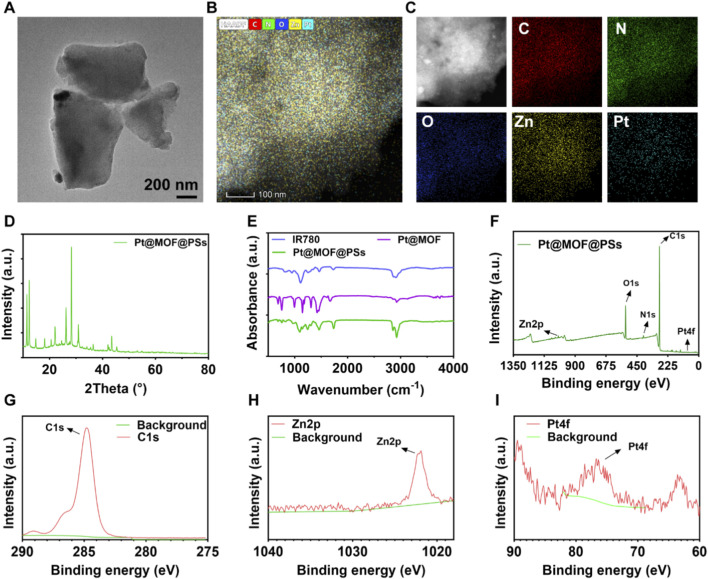
Characterization of Pt@MOF@PSs. **(A)** TEM images of Pt@MOF@PSs. **(B)** HAADF-STEM image. **(C)** Elemental distribution mapping. **(D)** XRD analysis. **(E)** FTIR spectrum. **(F)** XPS spectra. **(G)** High-resolution C1 s spectrum. **(H)** High-resolution Zn2p spectrum. **(I)** High-resolution Pt4f spectrum.

The Pt@MOF@PSs nanoplatform was fabricated by three steps. First, the IR780 dye was encapsulated within a liposomal formulation composed of Lecithin and cholesterol, resulting in IR780-loaded liposomes. A Pt-MOF nanozyme was then synthesized, and the IR780 liposomes were anchored onto it. The morphological features of Pt@MOF@PSs were visualized using electron microscopy (Figure 1A). Energy-dispersive spectroscopy (EDS) elemental mapping (Figures 1B,C) confirmed the presence of C, N, O, Pt, and Zn within the nanostructure. The crystalline structure of Pt@MOF@PSs was further confirmed by X-ray diffraction (XRD) analysis (Figure 1D). Fourier Transform Infrared (FTIR) spectroscopy revealed a characteristic absorption band at 758 cm^-1^, corresponding to C-H bending vibrations, which was linked to the IR780 dye (Figure 1E).

As shown in the UV-Vis absorption spectra (Supplementary Figure S1), IR780 exhibits a characteristic narrow absorption peak at approximately 780 nm. After successful loading of IR780, the resulting composite material Pt@MOF@PSs demonstrates significantly enhanced broad-spectrum absorption in the near-infrared region (650–900 nm), and its absorption peak is red-shifted compared to that of free IR780. This result confirms the successful incorporation of IR780 into the Pt@MOF framework and indicates a noticeable alteration in the absorption spectrum of the composite, which lays the foundation for efficient photothermal conversion and photodynamic effects under 808 nm laser excitation. X-ray photoelectron spectroscopy (XPS) analysis confirmed the incorporation of C, N, O, Pt, and Zn elements. High-resolution XPS spectra were further analyzed to determine the chemical states of C 1 s, Zn 2p, and Pt 4f orbitals, as depicted in Figures 1G–I. Dynamic light scattering (DLS) analysis was further employed to evaluate the hydrodynamic size distribution, polydispersity index (PDI), and surface charge of Pt@MOF and Pt@MOF@PSs (Supplementary Figure S2). Pt@MOF exhibited a moderate hydrodynamic diameter with a positive zeta potential (∼+15 mV), which can be attributed to the presence of the cationic surfactant CTAB used during synthesis. After surface modification, Pt@MOF@PSs showed an increased hydrodynamic size and a reversed surface charge (∼−10 mV), indicating successful coating with the negatively charged polymer/liposomal shell. Both formulations displayed relatively low PDI values (<0.2), suggesting good size uniformity. To further assess colloidal stability under biologically relevant conditions, the hydrodynamic sizes of Pt@MOF and Pt@MOF@PSs were measured in saline, DMEM, and FBS-containing medium. Pt@MOF and Pt@MOF@PSs maintained relatively stable size distributions across all tested conditions. These results confirmed the successful synthesis and structural characterization of the Pt@MOF@PSs nanozyme.

**FIGURE 2:**
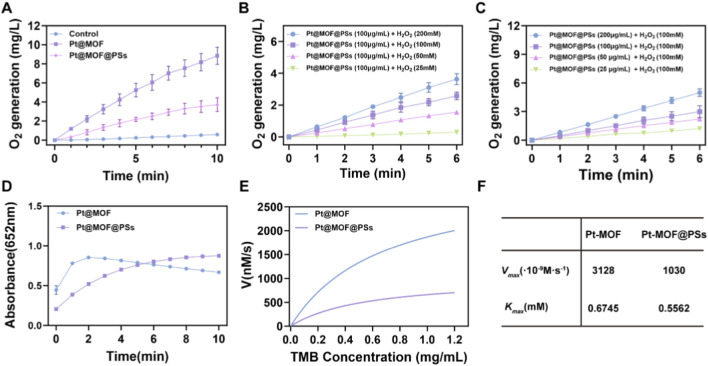
Catalytic activities of Pt@MOF@PSs. **(A)** Oxygen generation in different treatment groups. **(B)** Time-dependent oxygen generation at different H_2_O_2_ concentrations. **(C)** Oxygen production over time with different Pt@MOF@PSs concentrations. **(D)** POD-like activity measured using TMB over time. **(E)** TMB kinetics for POD-like activities. **(F)** TMB kinetics values.

The dual enzyme-like catalytic properties of Pt@MOF@PSs were further examined. As shown in Figure 2A, Pt@MOF@PSs produced a significant amount of oxygen over time, confirming its CAT-like activity. Pt@MOF showed a relatively stronger CAT-mimicking behavior compared to Pt@MOF@PSs, which may be due to partial blockage of catalytic sites by the IR780 liposomal coating. The CAT-like activity was also tested under different H_2_O_2_ concentrations. At a concentration of 100 μg/mL of Pt@MOF@PSs and with 200 mM H_2_O_2_, oxygen production reached 3.63 mg/L (Figure 2B). Moreover, the catalytic effect followed a concentration-dependent trend, with higher levels of Pt@MOF@PSs resulting in higher oxygen release (Figure 2C). Next, the POD-like activity of Pt@MOF@PSs was analyzed. Since POD enzymes convert H_2_O_2_ into cytotoxic ROS, the chromogenic substrate 3,3′,5,5′-tetramethylbenzidine (TMB), which turns blue in the presence of ROS, was used to measure activity. The absorbance spectra showed that with H_2_O_2_, Pt@MOF initially increased in absorbance at 652 nm, then declined after 2 min, while Pt@MOF@PSs showed a continuous increase over time (Figure 2D). To further analyze the catalytic performance, Michaelis-Menten kinetic analysis was performed using TMB as the substrate (Figure 2E). The maximum reaction velocities (V_max_) of Pt@MOF and Pt@MOF@PSs were calculated as 3.128 × 10^−6^ M s^−1^ and 1.030 × 10^−6^ M s^−1^, respectively. The Michaelis constants (K_m_), which indicate the substrate concentration required to reach half of V_max_, were found to be 0.6745 mM for Pt@MOF and 0.5532 mM for Pt@MOF@PSs (Figure 2F). In addition,the H_2_O_2_ concentrations used *in vitro* were chosen to ensure robust signal detection and reliable kinetic fitting; they do not directly represent endogenous tumor H_2_O_2_ levels, which are often reported in the tens of μM range and are highly heterogeneous. Accordingly, the obtained kinetic parameters (Km and Vmax) are intended to describe the intrinsic catalytic capability of the nanozyme and to support qualitative extrapolation (e.g., via the Vmax/Km ratio under low-substrate conditions), rather than to claim quantitative *in vivo* reaction flux. These results showed that both Pt@MOF and Pt@MOF@PSs possess effective CAT- and POD-mimicking properties, with Pt@MOF@PSs maintaining satisfactory catalytic efficiency despite liposomal modification.

Mechanistic note on dual enzyme-like behaviors. The relative predominance of catalase-like O_2_ generation versus peroxidase-like ROS production is condition-dependent (pH, H_2_O_2_ level, substrate availability, and subcellular localization). Given the mildly acidic extracellular pH of solid tumors and the more acidic endo/lysosomal compartments, Pt@MOF@PSs may display spatially differentiated catalytic behaviors, enabling O_2_ evolution in near-neutral regions and enhanced ROS generation in acidic microdomains. Considering that endogenous H_2_O_2_ is often reported in the tens of μM range in tumor cells/microenvironment, the catalytic rate *in vivo* is expected to be lower than that measured under high H_2_O_2_ *in vitro*; therefore, we frame the role of the nanozyme as a potential contributor to local oxygenation/oxidative stress enhancement rather than a guaranteed global TME reprogramming.

The photothermal effect of Pt@MOF and Pt@MOF@PSs solutions was measured using a thermal infrared camera. As shown in Supplementary Figure S3, the peak temperatures reached 38.7 °C and 48.9 °C for Pt@MOF and Pt@MOF@PSs, respectively, after 10 min of irradiation, indicating improved photothermal conversion efficiency of Pt@MOF@PSs, mainly due to IR780 liposomes. Photothermal stability was tested through four cycles of “heat up” and “cooling down” curves, showing consistent temperature profiles for both Pt@MOF and Pt@MOF@PSs (Supplementary Figure S4), which confirms their stable photothermal performance. Thus, Pt@MOF@PSs showed better photothermal conversion efficiency and stability, highlighting its potential for repeated PTT cycles.

**FIGURE 3:**
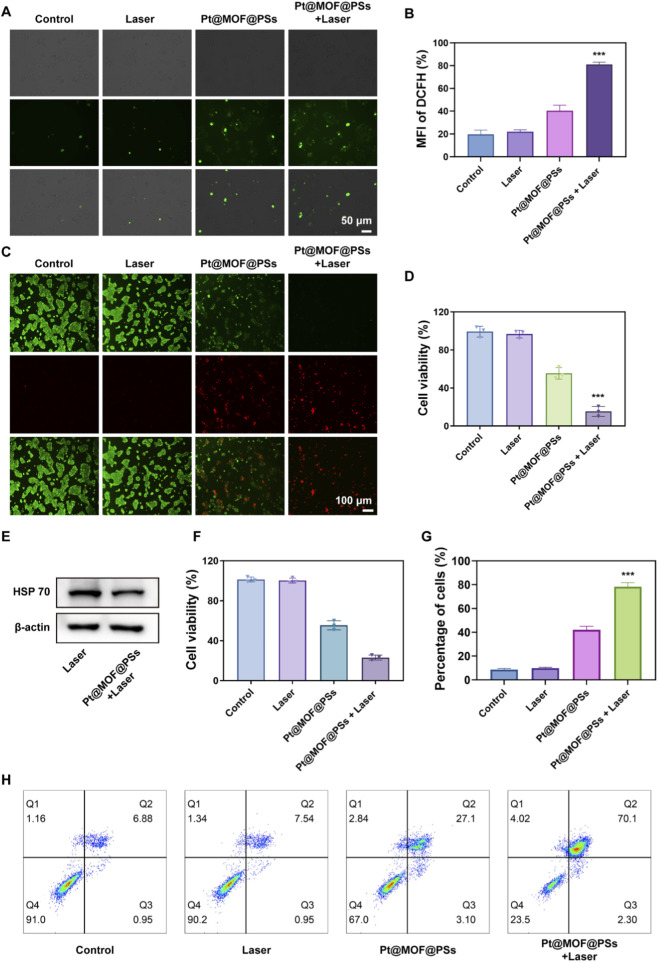
*In vitro* therapeutic efficacy of Pt@MOF@PSs. **(A)** Immunofluorescence staining of DCF to detect ROS generation. **(B)** Quantitative analysis of fluorescence intensity. **(C)** CLSM images from live/dead assay. **(D)** Quantification of live cell percentage. **(E)** Western blot analysis of HSP70 expression. **(F)** Cell viability by CCK8 assay. **(G)** Flow cytometry analysis of live cell proportion. **(H)** Representative flow cytometry plots. (n = 3, ns indicates P > 0.05, *P < 0.05,**P < 0.01, ***P < 0.001).

Based on its enzymatic properties, *in vitro* experiments were performed to investigate therapeutic efficacy against EC cells. ROS production was measured using the ROS probe 2,7-dichlorofluorescin diacetate (DCFH-DA). As shown in Figure 3A, minimal green fluorescence was observed in the control and laser-only groups. However, cells treated with Pt@MOF@PSs showed prominent green fluorescence, due to the POD-like activity of Pt@MOF@PSs. Under 808 nm laser irradiation, a significant increase in ROS generation was observed, further confirmed by mean fluorescence intensity (MFI) analysis of confocal images as a consequence of POD-like activity and PDT (Figure 3B). Live/dead cell staining was then performed using the Calcein AM/PI assay. As shown in Figures 3C,D, strong green fluorescence (live cells) with minimal red fluorescence (dead cells) was observed in both the control and laser-only groups. Treatment with Pt@MOF@PSs alone induced moderate cytotoxicity, resulting in 55.4% cell survival. In comparison, the Pt@MOF@PSs + laser group revealed intense red fluorescence, indicating extensive cell death. Moreover, Pt@MOF@PSs + laser treatment significantly decreased HSP70 protein levels, consistent with its photothermal effect (Figure 3E; Supplementary Figure S5). The antitumor efficacy was further confirmed using a CCK-8 assay. As shown in Figure 3F, consistent with live/dead staining, the Pt@MOF@PSs + laser group showed significant cytotoxicity. Flow cytometry analysis confirmed this effect, revealing early and late apoptosis rates of 1.88% and 74.2%, respectively, in the Pt@MOF@PSs + laser group (Figures 3G,H). Therefore, these results demonstrate that Pt@MOF@PSs has potent PTT-mediated cytotoxicity against EC cells after laser irradiation.

**FIGURE 4:**
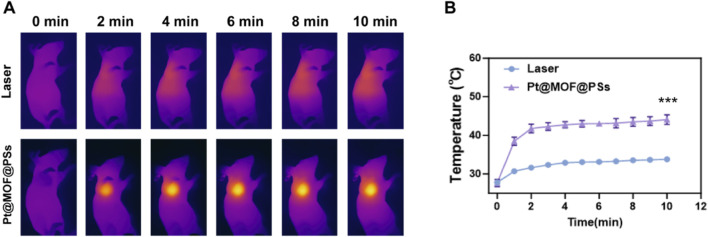
*In vivo* photothermal performance of Pt@MOF@PSs. **(A)** Thermal images of tumor-bearing mice administered with PBS or Pt@MOF@PSs under laser irradiation at different time points. **(B)** Temperature variation at tumor sites in all groups.

The photothermal effect of Pt@MOF@PSs was then tested in mice with EC tumors. After the tumors were established, the mice received intravenous injections of either PBS or Pt@MOF@PSs. Changes in tumor temperature under NIR laser irradiation were tracked using a thermal infrared camera (Figure 4A). As shown in Figure 4B, compared to the laser-only group, mice treated with Pt@MOF@PSs showed a significant temperature increase at the tumor site, reaching 44.1 °C, confirming the strong photothermal conversion ability of Pt@MOF@PSs for PTT. To test the hypoxia alleviation by Pt@MOF@PSs, tumors from mice in two groups were collected for HIF-1α staining. As shown in Supplementary Figure S6, the green signals representing hypoxia status was obviously reduced by Pt@MOF@PSs + Laser, verifying the hypoxia modulation.

**FIGURE 5:**
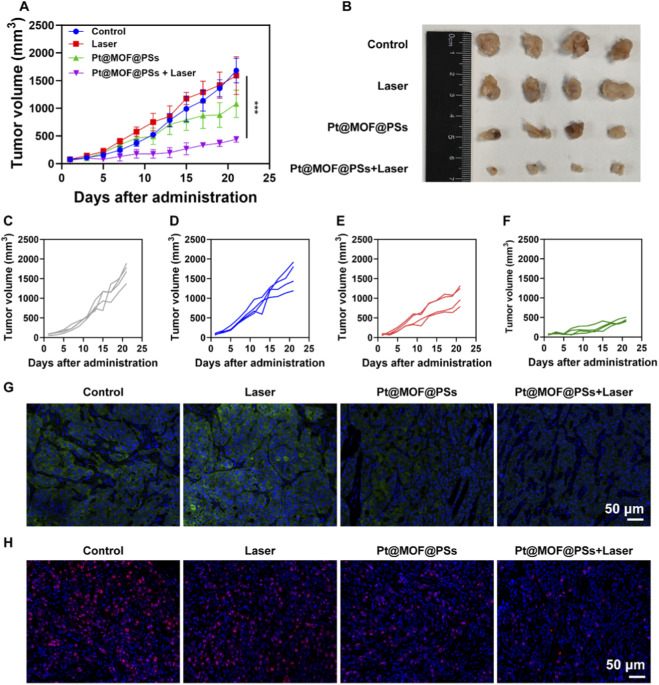
*In vivo* photothermal therapy on mice with EC tumors. **(A)** Tumor volume. **(B)** Macroscopic examination of tumors. Growth curves of tumor volume are shown in **(C)** the control group, **(D)** the laser group, **(E)** Pt@MOF@PSs, and **(F)** Pt@MOF@PSs + Laser. **(G)** Immunofluorescence staining of HSP70. **(H)** Immunofluorescence staining of Ki67. (n = 4, ns indicates P > 0.05, *P < 0.05,**P < 0.01, ***P < 0.001).

To evaluate the therapeutic efficacy of Pt@MOF@PSs in PTT, KYSE-150 tumor cells were subcutaneously implanted into the right flank of female nude mice. Four groups were established: (1) Control; (2) Laser only; (3) Pt@MOF@PSs; and (4) Pt@MOF@PSs + Laser. Tumor growth curves for each group are shown in Figure 5A, with detailed growth profiles of individual mice in Figures 5C–F. To further quantify the therapeutic efficacy, the tumor weights of mice from each treatment group were measured at the endpoint (Supplementary Figure S7). The results demonstrated that the control group exhibited the highest tumor weight, whereas the laser-only group showed no statistically significant difference compared to the control. Treatment with Pt@MOF@PSs alone significantly reduced the tumor weight. Notably, the Pt@MOF@PSs + laser combination group achieved the lowest tumor weight, with statistically significant differences observed compared to both the laser-only group and the Pt@MOF@PSs-alone group. The trend in tumor weight reduction was highly consistent with the tumor growth curves described earlier, collectively confirming the potent antitumor effect of Pt@MOF@PSs upon laser irradiation. Rapid tumor progression was observed in the control and laser-only groups. Treatment with Pt@MOF@PSs slowed tumor growth, primarily due to its CAT- and POD-like activities, which promoted the generation of cytotoxic ROS within the TME. When exposed to NIR laser irradiation, the Pt@MOF@PSs nanoplatform significantly suppressed tumor growth, due to the combined effects of PTT and enzyme-mimicking functions. Representative optical images (Figure 5B) further confirmed the strong tumor-eliminating ability of Pt@MOF@PSs + Laser treatment. HSP70, a key member of the heat shock protein family, plays a key role in maintaining cellular stability, regulating immune responses, and affecting tumor progression. When the local temperature in tumor tissue raises, the HSP70 upregulated and the antitumor effect might be impaired. Abundant ROS generation could downregulate the HSP70 level and thereby inhibit tumor growth. To study its modulation, tumor tissues collected at the end of the study were processed for HSP70 immunofluorescence staining. As shown in Figure 5G, strong green fluorescence signals, indicating high HSP70 expression, were observed in both the control and laser-only groups. Treatment with Pt@MOF@PSs decreased HSP70 expression, while the Pt@MOF@PSs + Laser group showed an almost complete suppression of HSP70 signals. Immunofluorescence staining of Ki67, a marker of cellular proliferation, was performed. Compared to the control, treatment with Pt@MOF@PSs resulted in a slight decrease in proliferative activity, whereas the Pt@MOF@PSs + Laser group showed a significant reduction in Ki-67 positive cells, indicating a potent antiproliferative effect (Supplementary Figure S8). These findings demonstrate that Pt@MOF@PSs effectively generate ROS to regulate the TME, and when combined with NIR laser-induced PTT, result in the potent suppression of tumor proliferation and the significant eradication of EC tumors.

**FIGURE 6:**
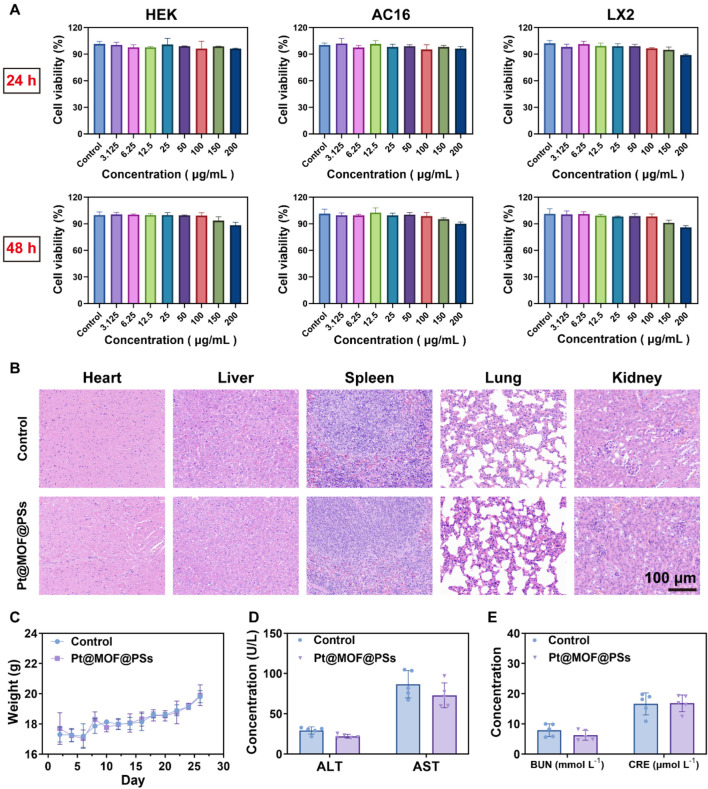
Biosafety analysis of Pt@MOF@PSs. **(A)** Cell viability of HEK, AC16, and LX2 cells after 24 and 48 h of treatment with different concentrations of Pt@MOF@PSs. **(B)** H&E staining of main organs. **(C)** Monitoring of body weight. **(D)** Liver function markers. **(E)** Kidney function markers.

Biosafety is an important factor in the clinical translation of nanotherapeutics. To evaluate the cytocompatibility of Pt@MOF@PSs, its effects were tested in multiple cell lines, including human embryonic kidney (HEK), immortalized human cardiomyocytes (AC16), and human hepatic stellate cells (LX2). Cells were exposed to different concentrations of Pt@MOF@PSs, and viability was measured using the CCK-8 assay after 24 and 48 h. As shown in Figure 6A, all 3 cell lines maintained viability above 90% at all concentrations and time points, indicating good cytocompatibility. Systemic toxicity was further evaluated in healthy nude mice administered Pt@MOF@PSs via tail vein injection. Body weight was monitored during the observation period, with no significant differences observed between treated and control groups (Figure 6C). At the study endpoint, major organs and blood samples were collected for histological and biochemical analyses. Histopathological examination showed no significant lesions or tissue damage in any of the main organs (Figure 6B). Liver and kidney function markers also remained within normal limits, confirming the absence of systemic toxicity (Figures 6D, E). These results showed that Pt@MOF@PSs has excellent biocompatibility and low toxicity both *in vitro* and *in vivo*, supporting its potential as a safe therapeutic nanoplatform.

## 3 Conclusion

This study concluded that esophageal cancer (EC) remains a significant global health challenge with limited effective treatment options. To address the limitations of conventional photothermal therapies (i.e., low photothermal conversion efficiency and poor tissue penetration), this study successfully developed a multifunctional nanozyme, Pt@MOF@PSs. This nanoplatform combines the catalytic ability of an MOF-based nanozyme with the photothermal properties of IR780, while also improving its water stability and tumor-targeting ability. After accumulating in the TME, Pt@MOF@PSs exerted a synergistic therapeutic effect by reducing hypoxia, generating ROS through H_2_O_2_ consumption, and producing strong photothermal effects under NIR laser irradiation. These combined actions effectively reprogrammed the resistant TME, leading to increased eradication of esophageal squamous cells and significant tumor suppression. Therefore, this study highlights Pt@MOF@PSs as a promising strategy that combines catalytic and photothermal therapies, offering a potential advancement in the treatment of EC.

## Data Availability

The raw data supporting the conclusions of this article will be made available by the authors, without undue reservation.
