# Supplementary material for: Synergistic photothermal therapy of esophageal cancer using Pt@MOF@PSs nanozymes
Source: Front Bioeng Biotechnol. 2026 Feb 5;14:1729547. doi: 10.3389/fbioe.2026.1729547 (PMC12916567; doi:10.3389/fbioe.2026.1729547)
Supplement: Supplementary file 1 [file Supplementaryfile1.docx]

**Synergistic Photothermal Therapy of Esophageal Cancer using Pt@MOF@PSs Nanozymes**

Yuhang Shang^1^, Yujie Zhao^2^, Ran Ding^3^, Xinyue Gao^1^, Qi Li^1^, Ziyi Li^1*^, Xinglan An^1*^

^1^Key Laboratory of Organ Regeneration and Transplantation of the Ministry of Education, The First Hospital of Jilin University, Jilin University, Changchun, China. 130033.

^2^ Department of Intensive Care Unit, The First Affiliated Hospital of Jiamusi University, Jiamusi, China. 154000.

^3^Candidate State Key Laboratory of Pharmaceutical Biotechnology and Jiangsu Key Laboratory of Molecular Medicine, Nanjing University Medical School, Nanjing, China. 210093.

Email: [ziyi@jlu.edu.cn](mailto:ziyi@jlu.edu.cn) (Z. Li); [anxinglan@jlu.edu.cn](mailto:anxinglan@jlu.edu.cn) (X. An)

**Experimental Section**

1. **Chemicals and Materials**

2-methylimidazole and Zn(NO_3_)_2_·6H2O were purchased from Sigma-Aldrich. Hexadecyl trimethyl ammonium bromide (CTAB) was purchased from Adamas. NaBH_4_, acetic acid and sodium acetate were purchased from Macklin. RPMI 1640 and FBS were purchased from Gibco. Cell counting kit-8 (CCK-8) was purchased from APExBIO. 2’, 7’-dichlorodihydrofluorescein diacetate (DCFH-DA) was purchased from Beyotime. Annexin V-FITC/PI Apoptosis Detection Kit was purchased from Yeasen. Acetoxymethylester of calcein (Calcein-AM), propidium iodide (PI) were purchased from Biosharp. Anti-Ki67 antibody and anti-HSP70 antibody were purchased from Servicebio. All aqueous solutions were prepared using deionized (DI) water purified by a Direct-Q® 3 water purification system (Millipore, USA).

1. **Fabrication of Pt-MOF@PSs**

Synthesis of Pt-MOF：Under stirring at 500 rpm, 200 μL of chloroplatinic acid solution (10 mg) was added to a mixture containing 550 μM CTAB in 1.75 mL of 2-methylimidazole (790 mM), followed by the addition of 0.25 mL of an aqueous solution of Zn(NO₃)₂·6H₂O (97.5 mM). After stirring for 5 minutes, 200 μL of NaBH₄ (1 mg/mL) was added dropwise. The mixture was then stirred for 3 hours and subsequently allowed to stand at room temperature for another 3 hours. Finally, the prepared Pt-MOF was collected by centrifugation at 3500 g for 10 minutes, washed twice with water, and vacuum freeze-dried.

Synthesis of Pt-MOF@PSs：Dissolve lecithin (10 mg), cholesterol (2 mg), and DSPE-PEG-IR780 (1 mg) in 3 mL of chloroform/ethanol (v/v = 2:1), and remove the solvents completely under vacuum. Subsequently, add 2 mg of Pt-MOF nanozyme and perform ultrasonication at 200 W for 10 minutes. After collecting and washing three times, the Pt-MOF@PSs liposomal nanozyme system is obtained

1. **Enzymic-mimicking activities measurements**

Evaluation of Catalase (CAT)-like Activity：The dissolved oxygen meter (Lei-ci, China) was used to monitor the oxygen generation in the reaction system of nanozymes (Pt@MOF, Pt-MOF@PSs) with H_2_O_2_ at room temperature. The oxygen production was monitored for the same concentration of Pt-MOF@PSs (100 μg/mL) with different concentrations of H_2_O_2_ (25-200 mM), and for different concentrations of Pt-MOF@PSs (25-200 μg/mL) with the same concentration of H_2_O_2_ (100 mM). The monitoring time was 10 minutes. The specific procedure was as follows: under pH 4.5 conditions, the aqueous solution of the nanozyme was mixed with H_2_O_2_, and the amount of oxygen generated was recorded every 1 min using the dissolved oxygen meter.

Evaluation of Peroxidas (POD)-like Activity: The POD-like activity of the nanozyme was assessed using TMB as the chromogenic substrate. Specifically, under pH 4.5 conditions, the nanozyme, H_2_O_2_, and TMB were mixed, and the change in absorbance of the reaction system was immediately monitored at 652 nm. The peroxidase-like activity of Pt-MOF@PSs was subsequently evaluated using the Michaelis-Menten equation, by analyzing the reaction rates at varying TMB concentrations.

1. **Cell line and cell culture**

The human esophageal cancer cell line (EC109) was purchased from Procell Life Science & Technology Co., Ltd. and cultured in Roswell Park Memorial Institute 1640 Medium (RPMI 1640) supplemented with 10% fetal bovine serum (FBS) and 1% penicillin/streptomycin at 37°C under 5% CO₂。

1. **Cell viability assessment**

Liver cells (LX-2), cardiomyocytes (AC16), and kidney cells (HEK293) were seeded on a 96-well plate at a density of 5,000 cells per well and incubated for 24 hours. Subsequently, the cells were treated with varying doses of Pt-MOF@PSs (0-200 μg/mL). After 24 and 48 hours of treatment, cell viability was assessed using the Cell Counting Kit-8 (CCK-8) assay

1. **Hemolysis rate measurement**

Red blood cells (RBCs) were separated from serum by centrifugation at 1500 rpm for 10 minutes. Subsequently, different concentrations of Pt-MOF and Pt-MOF@PSs (ranging from 12.5 to 400 μg/mL) were incubated with the RBCs at 37°C for 30 minutes (the materials were diluted with PBS). Finally, the above mixture was centrifuged, and 100 μL of the supernatant was collected to measure its absorbance at 578 nm using a microplate reader (Infinite F50, Switzerland).

Hemolysis ratio=[OD_(sample)_-OD_(PBS)_]/[_OD(ddH2O)_-OD_(PBS)_] ×100%

1. **ROS detection**

Intracellular ROS levels were measured using 2′,7′-dichlorofluorescin diacetate (DCFH-DA) staining. EC109 cells were seeded in 6-well plates at a density of 15,000 cells per well and incubated for 24 hours. The esophageal cancer cells were then subjected to the following treatments: light irradiation only, Pt-MOF@PSs treatment only, and a combination of Pt-MOF@PSs and light irradiation. After 24 hours of treatment, the cells were washed with PBS to remove any free Pt-MOF@PSs. Finally, the cells were imaged using an inverted fluorescence microscope (EVOS M5000, USA) (emission wavelength: 520±20 nm; excitation wavelength: 488 nm).Additionally, cells treated with the same methods were collected, stained with DCFH-DA, incubated at 37°C for 30 minutes, and washed three times with PBS. The collected cells were then analyzed by flow cytometry (BD Biosciences, USA).

1. **Live/dead assay**

EC109 cells were seeded in 6-well plates at a density of 15,000 cells per well and incubated for 24 hours. The esophageal cancer cells were then subjected to the following treatments: light irradiation only, Pt-MOF@PSs treatment only, and a combination of Pt-MOF@PSs and light irradiation. Subsequently, cell viability was assessed using Calcein AM (1 μM) and propidium iodide (PI) at 2 μM (Calcein AM for live cells; PI for dead cells). Fluorescence images were captured using an inverted fluorescence microscope. Additionally, apoptosis of EC109 cells was evaluated using an Annexin V apoptosis detection kit. Cells treated with the same methods were collected, washed three times with cold PBS, and then stained with Annexin V and PI. Finally, the collected cells were analyzed by flow cytometry

1. **Animal model**

The study was approved by the Animal Ethics Committee of the First Hospital of Jilin University (Approval No. JDYY20250927). The experiments were conducted under the institutional animal care license SYXK(Ji) 2024-0022. The ethical approval was granted on September 8, 2025. All procedures strictly adhered to the "Guide for the Care and Use of Laboratory Animals," with a focus on minimizing animal suffering and ensuring welfare.

EC109 cells cultured in vitro were digested with trypsin, collected, and kept on ice. The cells were then resuspended in a certain amount of sterile PBS and mixed with 20% Matrigel to adjust the final cell concentration to 1 × 10⁷ cells/mL. The cell suspension was subcutaneously inoculated into the right dorsal region of female BALB/c-nu nude mice at a dose of 100 μL per mouse. The injection site was observed for the formation of a raised bulge. Tumor size was measured using a caliper by recording the length and width of the tumor, and the tumor volume was calculated using the formula: V = length × width × width / 2

1. **Photothermal ability evaluation in vitro and in vivo**

Two nanozymes solutions (Pt@MOF and Pt-MOF@PSs) with a concentration of 100 μg/mL were placed in 1.5 mL centrifuge tubes, respectively. Subsequently, an 808 nm laser emitter (with a laser power intensity of 1 W/cm2) was used to irradiate the solutions to record the temperature increase of each system within 600 s. PBS was used as a control in this process. The 808 nm laser emitter (MDL-III-808, China) (with a laser power intensity of 1 W/cm2) was also employed to irradiate the test solutions (Pt@MOF and Pt-MOF@PSs) at 100 μg/mL for multiple cycles. Each cycle consisted of a heating period and a natural cooling period to evaluate their photothermal stability.

Pt-MOF@PSs (at the dosage of 15 mg/kg) was administered to EC109 tumor-bearing mice via tail vein injection. The tumor area was exposed to an 808 nm laser emitter for continuous irradiation over a period of 10 min. Images and the temperature elevation curve of the tumor site were acquired using an infrared thermal imaging camera (FLIR A655sc, USA).

1. **Tumor inhibition evaluation**

Tumor-bearing nude mice were randomly divided into 4 groups: (1) PBS (200 μL), (2) PBS (200 μL) + laser irradiation, (3) Pt-MOF@PSs (15 mg/kg), and (4) Pt-MOF@PSs (4 mg/kg) + laser irradiation. All mice received intravenous injections via the tail vein. At 12 hours post-injection, the tumors were irradiated with an 808 nm laser at a power density of 1 W/cm² for 10 minutes. The temperature changes at the tumor site were monitored and recorded in real-time using an infrared camera to obtain infrared thermal images. Throughout the 21-day treatment period, tumor sizes were measured every two days. After euthanasia, the tumors were collected and subjected to immunofluorescence staining for Ki67 and HSP70. All animal experiments were conducted in accordance with the protocol approved by the Animal Ethics Committee of the Experimental Animal Center of Jilin University (No. SYXK (Ji) 2024-0022). All procedures followed the guidelines outlined in the “Guide for the Care and Use of Laboratory Animals”.

1. **In vivo toxicity assessment.**

Pt-MOF@PSs (20 mg/kg) and PBS were intravenously injected into two groups of mice (n=5 per group) twice a week. After 21 days, organ and blood samples were collected from both groups. Subsequently, the heart, liver, spleen, lungs, and kidneys of the mice were subjected to H&E (Hematoxylin and Eosin) staining, and blood parameters were measured for both groups. The relevant blood parameters analyzed included Alanine Aminotransferase (ALT), Aspartate Aminotransferase (AST), Creatinine (CRE), and Blood Urea Nitrogen (BUN).

**13. Statistical analysis**

Data are presented as the mean ± standard deviation (SD) from at least three independent replicates. Statistical analysis involved two-sided Student t-test for two groups and one-way ANOVA for multiple groups. *P<0.05 was considered to be statistically significant.


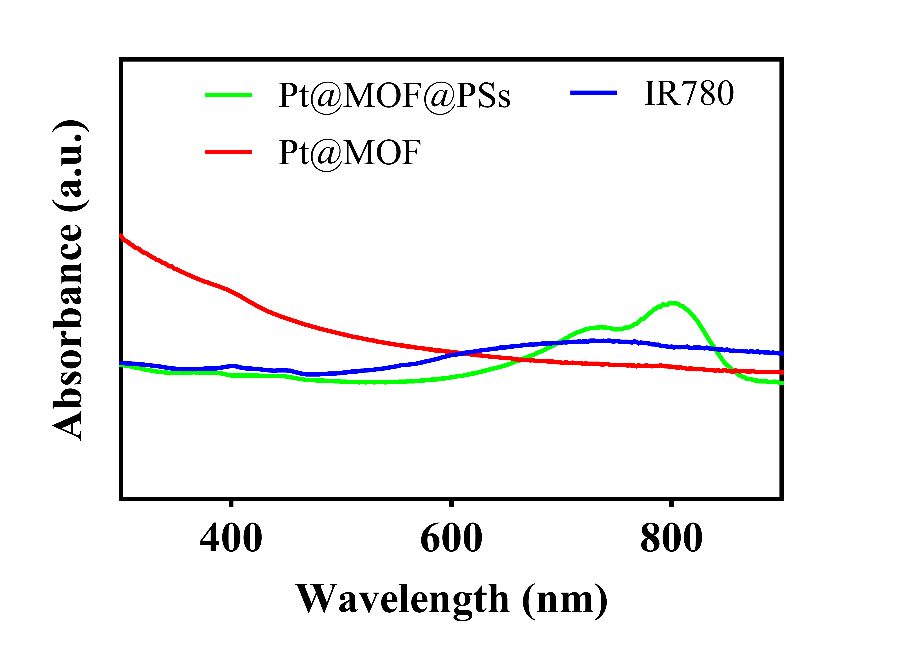


Figure S1. UV-vis spectra of IR780, Pt@MOF and Pt@MOF@PSs.


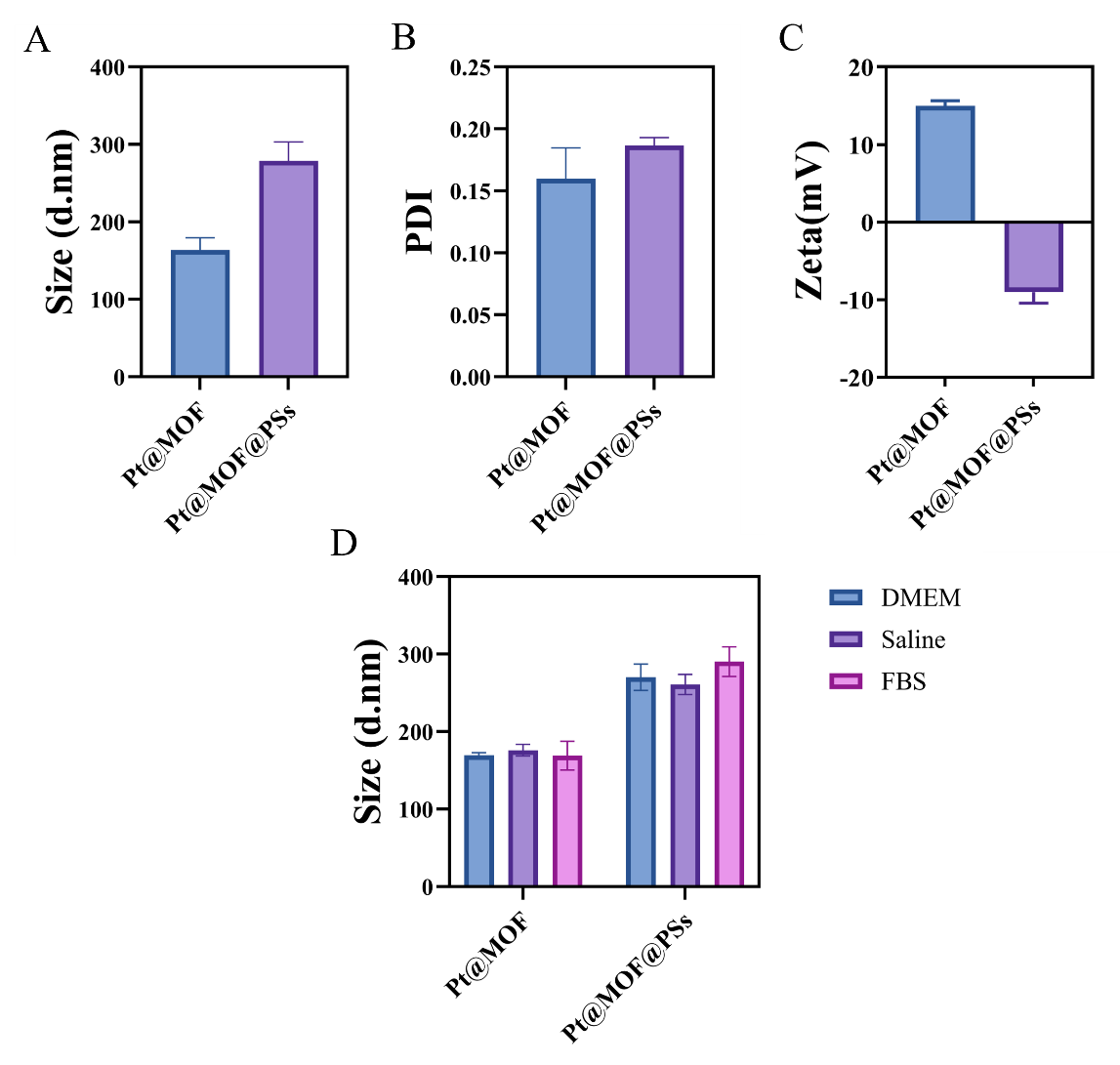


Figure S2. Physicochemical characterization and colloidal stability of Pt@MOF and [Pt@MOF@PSs. (A)](mailto:Pt@MOF@PSs.(A)) Hydrodynamic diameter of Pt@MOF and Pt@MOF@PSs measured by dynamic light scattering (DLS). (B) Polydispersity index (PDI) of Pt@MOF and Pt@MOF@PSs. (C) Zeta potential of Pt@MOF and Pt@MOF@PSs. (D) Hydrodynamic size of Pt@MOF and Pt@MOF@PSs dispersed in different media (saline, DMEM, and FBS-containing medium), demonstrating improved colloidal stability of Pt@MOF@PSs in biologically relevant environments. Data are presented as mean ± SD (n = 3).

**
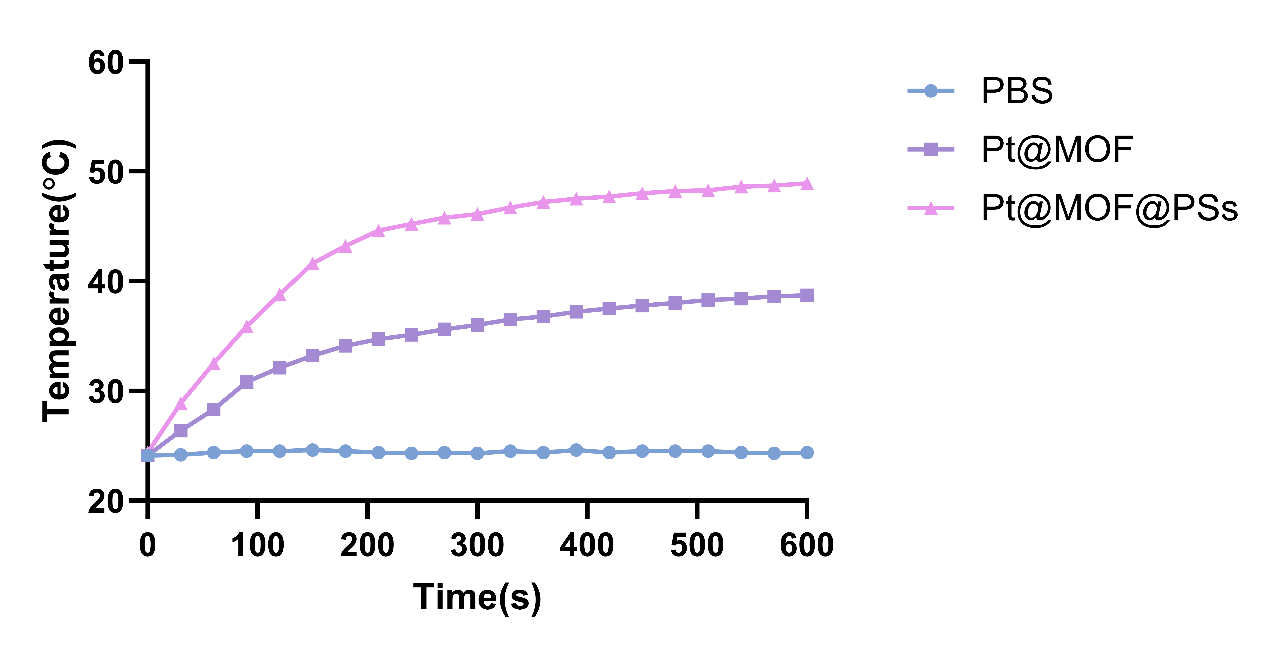
**

Figure S3. Temperature change curve of various groups under 808 nm laser irradiation.


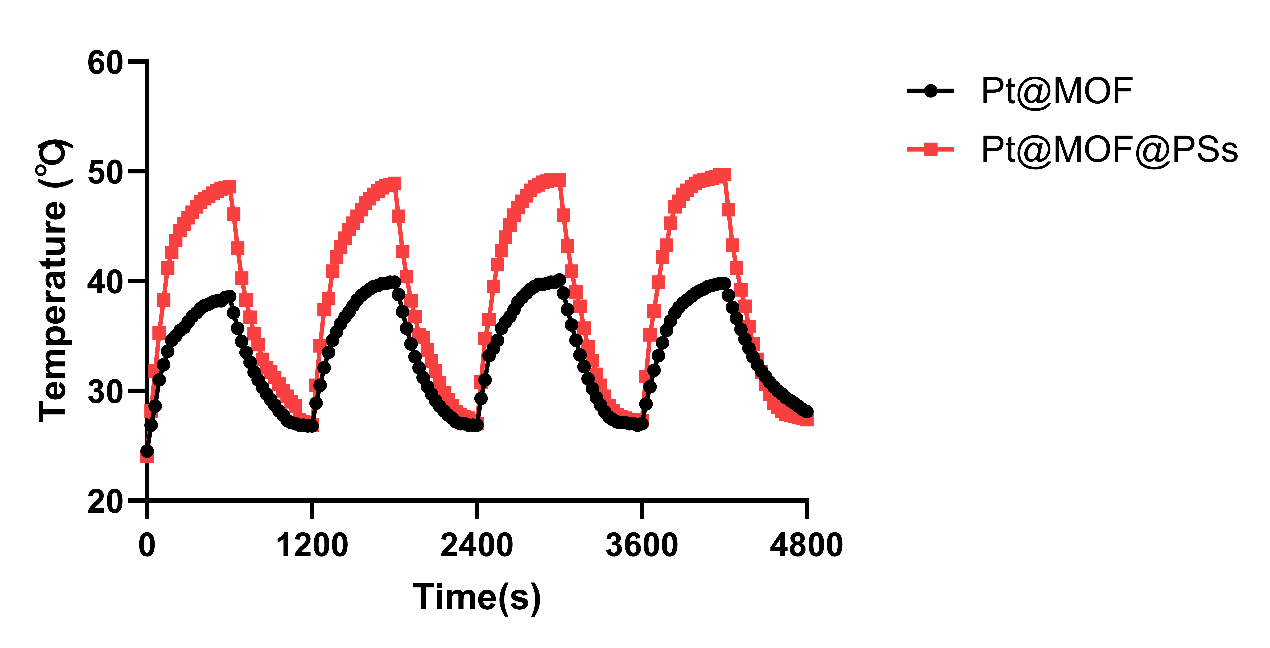


Figure S4. Heating and cooling down curves of various groups.


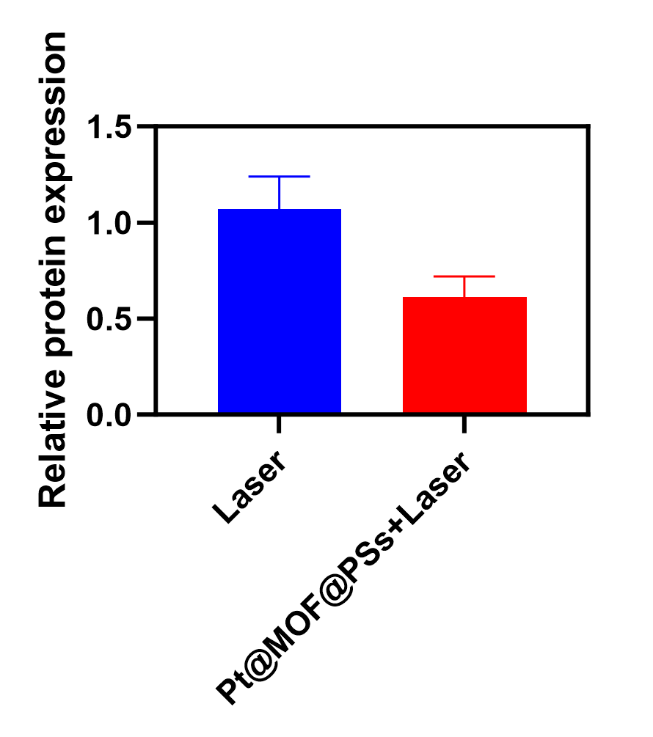


Figure S5. Relative protein expression of HSP70.


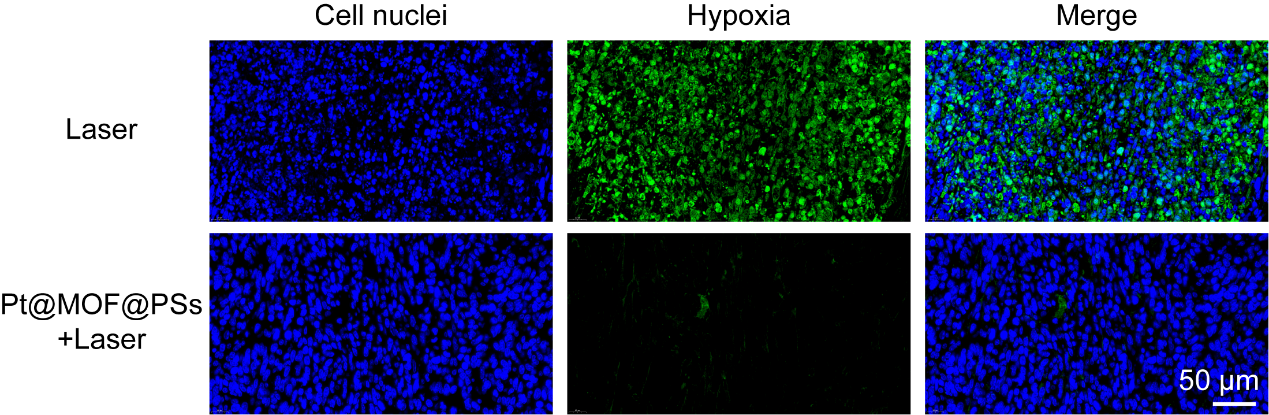


Figure S6. Immunofluorescence staining of HIF-1α.


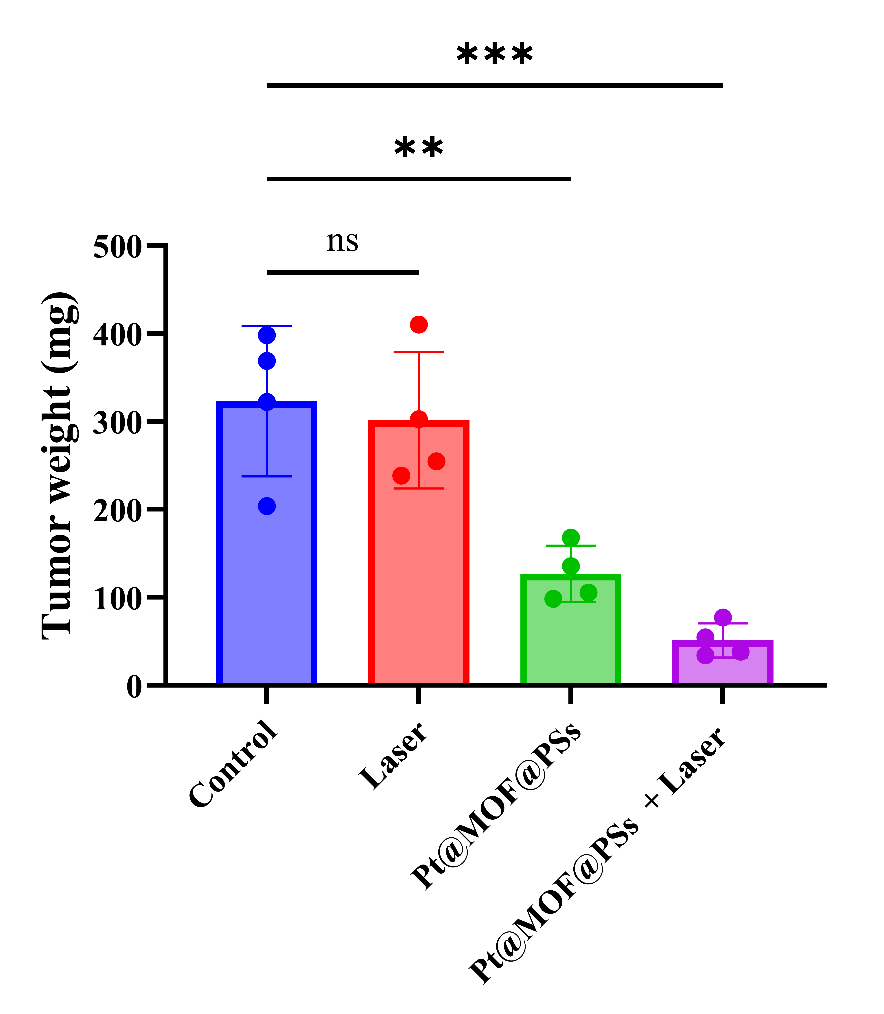


Figure S7. The average weight of tumors harvested at the end of these treatments. (n=4, ns indicates P > 0.05, **P < 0.01, ***P< 0.001).


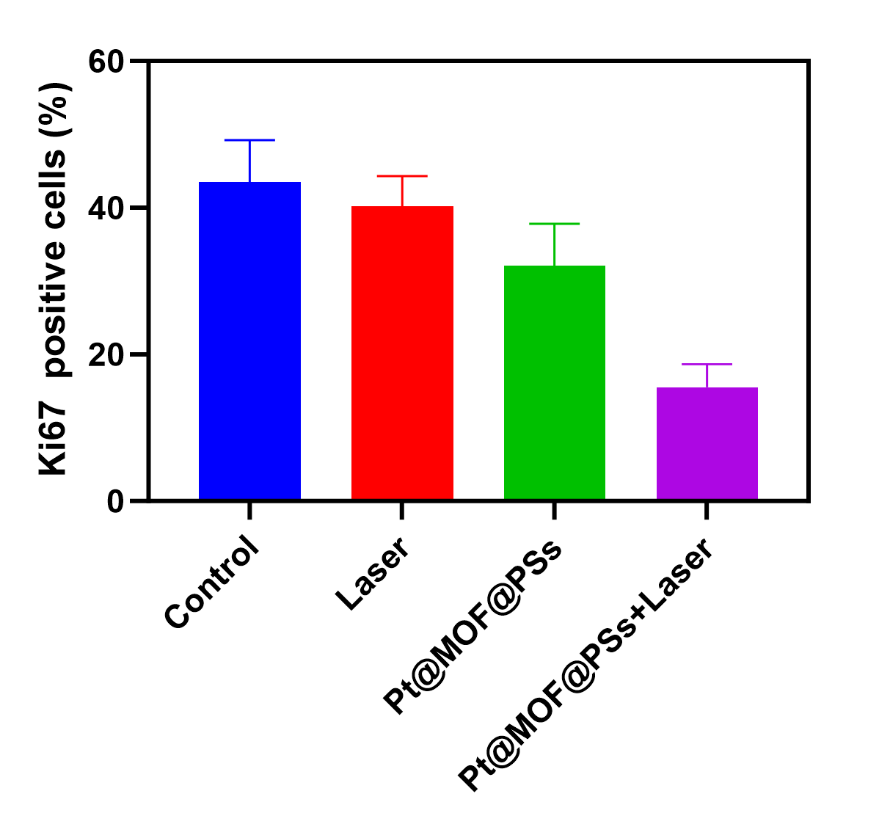


Figure S8. Ki67 positive cells quantification.
